# Species-Specific Differences in the Microbiomes and Organic Exudates of Crustose Coralline Algae Influence Bacterioplankton Communities

**DOI:** 10.3389/fmicb.2019.02397

**Published:** 2019-11-08

**Authors:** Zachary A. Quinlan, Raphael Ritson-Williams, Brenna J. Carroll, Craig A. Carlson, Craig E. Nelson

**Affiliations:** ^1^Department of Oceanography, Daniel K. Inouye Center for Microbial Oceanography: Research and Education, University of Hawai‘i at Mānoa, Honolulu, HI, United States; ^2^Department of Biology, San Diego State University, San Diego, CA, United States; ^3^Hawai‘i Institute of Marine Biology, University of Hawai‘i at Mānoa, Honolulu, HI, United States; ^4^Department of Ecology, Evolution and Marine Biology, University of California, Santa Barbara, Santa Barbara, CA, United States; ^5^Sea Grant College Program, University of Hawai‘i at Mānoa, Honolulu, HI, United States

**Keywords:** crustose coralline algae, dissolved organic matter, macroalgae, microbiome, coral reef ecology, 16s rRNA gene amplicon sequencing

## Abstract

Crustose coralline algae (CCA) are critical members of the coral reef ecosystem, yet they remain poorly studied. Recent research on CCA has shown that only a few species play a significant role in the settlement of coral larvae through either the production of chemical settlement cues or the facilitation of specific microbial communities that are hypothesized to influence coral settlement. Thus, defining how DOM exudates differ between CCA species and the bacterioplankton communities these exudates facilitate is important for understanding the role of CCA in invertebrate settlement. We conducted single day exudation experiments on two species of CCA to compare tissue microbiome community structure, DOM production and the effect of DOM on the bacterioplankton community. We collected exudates from *Hydrolithon reinboldii* and *Porolithon onkodes* in both filter-sterilized seawater and unfiltered seawater from Kāne‘ohe Bay, Hawai‘i. Our results demonstrate that while both species exude equivalent quantities of dissolved organic carbon they differ in the composition of fluorescent DOM and fostered distinct microbial communities. *P. onkodes* exudates facilitate more microbial OTUs associated with coral disease, whereas *H. reinboldii* facilitated OTUs known to produce antimicrobial compounds. Our results highlight species-specific differences in the composition of fDOM exudates of CCA and the effect of those on microbial community structure.

## Introduction

Crustose coralline algae (CCA) are cryptic components of almost every reef habitat. These encrusting red algae (Rhodophyta, order Corallinales) perform multiple ecosystem services on reefs from binding loose substrata together (Littler and Littler, 2013) to building large reef structures (Adey, 1975). There are more than 1600 described species of CCA globally but the taxonomic characters used to differentiate them typically require a microscope and considerable expertise. Indeed the current species diversity is probably an underestimation due to cryptic lineages (Gabrielson et al., 2018). With such species diversity there is high probability of diverse niches and adaptations for survival in marine habitats, however, there remain relatively few studies comparing what effect the different CCA species physiologies have on their environments.

One of the best studied ecological roles of CCA is as settlement facilitators for coral larval settlement (Heyward and Negri, 1999; Raimondi and Morse, 2000). However, the mechanism for cueing settlement is still unclear. Early research found that coral settlement would occur on the dead skeleton of CCA (Heyward and Negri, 1999). However, more recently, a few studies have shown that bacterial strains isolated from CCA are capable of inducing coral larval settlement without the CCA present (Negri et al., 2001; Webster et al., 2004; Tran and Hadfield, 2011; Sneed et al., 2014). This suggests two different mechanisms of settlement facilitation, one being facilitating compounds in the cell walls of the CCA (Morse and Morse, 1991; Kitamura et al., 2007) and the other a compound produced by bacteria (Tebben et al., 2011; Sneed et al., 2014). These two hypotheses are not mutually exclusive, in fact it is highly likely the settlement response is a result of the interactions between exudates, tissue bound DOM and associated microbial communities. Although CCA does have specific associated microbial communities (Barott et al., 2011; Webster et al., 2011, 2013), these communities are not ubiquitous and do differ by CCA species (Sneed et al., 2015).

Moreover, research is showing that coral larvae have settlement preferences among different species of CCA (Harrington et al., 2004; Ritson-Williams et al., 2010, 2014), with some species of CCA facilitating and others inhibiting settlement for most of the coral species tested so far (Ritson-Williams et al., 2016). These preferential settlement patterns are possibly a result of the differing mechanisms by which CCA suppress the growth of opportunistic biofilms and macroalgae such as the production of secondary metabolites (Gomez-Lemos and Diaz-Pulido, 2017) and mechanical sloughing of epithelial tissue (Keats et al., 1997). Indeed, coral larval settlement studies have shown higher levels of sloughing and thus hazardous to larvae in *Porolithon onkodes* relative to other species of CCA such as *Hydrolithon reinboldii* and *Titanoderma prototypum* (Harrington et al., 2004).

Recent research has shown that CCA exudes dissolved organic carbon (DOC; Haas et al., 2011, 2013; Mueller et al., 2014) and that coral rubble, partially encrusted by CCA, produces fluorescent dissolved organic matter (fDOM) compositionally distinct from corals and macroalgae (Quinlan et al., 2018). CCA specific compounds can also remain tissue bound (Tebben et al., 2015) in the form of glycoglycerolipids and polysaccharides extracted from the cell walls of CCA (Tebben et al., 2015) as well as tetrabromopyrrole extracted from *Pseudoalteromonas* (Tebben et al., 2011; Sneed et al., 2014). These compounds have been shown to act directly as cues for coral larvae metamorphosis (Kitamura et al., 2009; Tebben et al., 2015). However, the extracellular DOM could be remineralized by bacterioplankton, facilitating specific microbial communities (Nelson et al., 2013) which may then act as the cue for metamorphosis (Negri et al., 2001). To date the effect of extracellular organics from different CCA taxa on microbial community structure has not been tested and could directly affect the ability of the CCA species to induce settlement. Moreover, it remains unknown to what degree CCA species differ in exudate composition.

The present study compared the exudates, tissue bound and water column associated microbial communities from two species of CCA belonging to genera that have been previously shown to have different effects on coral larval settlement, *H. reinboldii* and *P. onkodes* (facilitating and inhibiting, respectively; Harrington et al., 2004). Our goal was to measure the quantity and quality of DOM produced by the different CCA, the response of bacterioplankton communities to the DOM released and the release of CCA-associated microbes into the water through sloughing. We hypothesized that (1) *H. reinboldii* would produce exudates more similar to coral (more labile humic-like fDOM and more proteinaceous fDOM; Quinlan et al., 2018), (2) *H. reinboldii* would facilitate growth of more beneficial microbial communities whereas *P. onkodes* would foster opportunistic pathogens and (3) *P. onkodes* would slough more tissue and thus more bacteria into the water. By assessing fDOM production, microbial community structures and growth rates of both the microbiome and bacterioplankton we begin to tease apart differences in these CCA species which might be related to coral larval settlement facilitation and inhibition.

## Materials and Methods

### Crustose Coraline Algae Collection and Identification

Both *H. reinboldii* and *P. onkodes* were collected from Patch Reef 42 (21.4785°, –157.8281°) in Kāne‘ohe Bay, O‘ahu, Hawai‘i on 4 May 2017. *P. onkodes* is a common CCA species in the Pacific Ocean that is often used for larval settlement experiments with coral species in Australia (Heyward and Negri, 1999). It is typically found in high light and high flow environments, such as at the top of the patch reefs in Kāne‘ohe Bay. This species is characterized by its smooth surface texture, and diagnostic depressions of trichosite fields. While there is a recent paper showing that this species is a species complex globally (Gabrielson et al., 2018), we retain the use of the name *P. onkodes* here to be consistent with the published taxonomic monograph for CCA in Hawai‘i (Adey et al., 1982). *H. reinboldii* is also a common CCA species that is found throughout the Pacific Ocean. It is known to facilitate coral larval settlement (Harrington et al., 2004). This species often lives cryptically in cracks in the reef or on the bottom of small pieces of calcium carbonate rubble. It is characterized by slightly raised hemispherical single pore conceptacles (400–600 μm in diameter), and a patchy surface texture referred to as tessellate (Adey et al., 1982).

Fragments of both species of CCA were trimmed using bone cutters to ensure only a single plant was on each fragment. Each fragment still retained bare calcium carbonate along with the individual species of CCA. To control for the bare calcium carbonate, encrusted fragments of calcium carbonate were similarly trimmed to remove any small CCA plants and epiphytes leaving only the calcium carbonate rubble and endophytes. After fragmentation the specimens were haphazardly placed into six containers and randomized within a 1300 L flow through seawater bath to maintain all treatments at a stable temperature, which was the same as those found in Kāne‘ohe Bay. As there are currently no studies on the effect of fragmentation on exudate production we allowed the fragmented algae to recover for 5 days before starting the exudation experiment. Flow through seawater baths were covered by shade cloth to reduce natural irradiance to levels similar to those found at depth in Kāne‘ohe Bay where both species are naturally found. Both species were exposed to the same light levels as to not bias the results by variation of abiotic parameters.

### Incubations and Sample Collection

Twenty-four 250 mL glass beakers were washed with 10% volumetric HCl, rinsed with milliq-water and air-dried. At 07:30 on 9 May, 3 L of seawater (sand filtered and collected from the Hawai‘i Institute of Marine Biology flow-through seawater system in Kāne‘ohe Bay) was vacuum pre-filtered through 0.2 μm polyethersulfone filters (47 mm; Sterlitech) in a 500 mL polysulfone graduated filter holder. Before water was aliquoted into the beakers, samples for fluorescent DOM (fDOM), dissolved organic carbon (DOC), and flow cytometry (FCM) were collected from the 500 mL polysulfone graduated filter holder. Each beaker was filled and randomized within a 1300L flow through seawater bath to maintain stable temperature between the treatments. Each organism treatment beaker (water control, calcium carbonate control, *H. reinboldii*, or *P. onkodes*) was filled with seawater (filtered or unfiltered) and replicated (*n* = 3) for a total of 24 beakers (4 organismal treatments ^∗^ 2 water treatments ^∗^ 3 replicates). Filtered and unfiltered treatments were designed to capture differences in sloughing behavior between species. Multiple trimmed fragments of each organism were placed within their respective beakers so that the total surface area within each replicate beaker was standardized to 20–30 square cm (25.57 ± 4.13 cm^2^). The incubation began at 9:00 and was halted at 17:00 to maintain only exudates produced during the daylight hours. Surface area was digitally determined at the end of the experiment by analyzing images to scale with image-J (Schindelin et al., 2012).

DOM samples were collected at the beginning of the experiment before aliquoting the water at 9:00 and from each beaker at 17:00. DOM samples were immediately filtered through a 0.2 μm polyethersulfone filter (47 mm; Sterlitech) in a 500 mL polysulfone graduated filter holder. Filtrate was poured directly from polysulfone graduated filter holder into its respective sample vial, Filtrate for fDOM samples were collected in acid washed, combusted, triple sample-rinsed amber borosilicate vials with Teflon septa caps and stored dark at 4°C until analysis for fDOM within 24 h. DOC was collected in acid washed, combusted, triple sample rinsed clear borosilicate vials with Teflon septa caps and measured as non-purgeable organic carbon via acidification, sparging and high temperature platinum catalytic oxidation on a Shimadzu TOC-V at the UCSB DOM Analytical Lab following the methods outlined by Carlson et al. (2010). Samples for flow cytometry were collected by pipet (1 ml amended to a final concentration of 0.5% paraformaldehyde, mixed by inversion, snap frozen −80°C) at 9:00, 13:00, and 17:00.

### Sample Analysis

#### Flow Cytometry

Flow cytometry was used to measure total nucleic acid-stained cell concentrations. Samples were thawed and 200 μL were aliquoted into u-bottomed 96-well autosampler plates and stained with 2 μL of 100X SYBR Green I stain (final concentration of 0.5X). Samples were analyzed on an Attune Acoustic Focusing Cytometer with Autosampler Attachment (Life Technologies, Eugene, OR, United States). Samples were run at a flow rate of 100 μL min^–1^ on standard sensitivity; 150 μL of sample was aspirated, 75 μL was counted and data was collected only from the last 50 μL (event rates were empirically determined to be steady only after 25 μL of continuous sample injection per Nelson et al., 2015).

#### Fluorescence Spectroscopy

Samples for fluorescence spectroscopy were measured using an Horiba Aqualog scanning fluorometer following the methods of Nelson et al. (2015), including scan time and resolution, spectral data processing, inner filter correction, Raman unit standardization, blank subtraction and PARAFAC modeling (Stedmon and Bro, 2008; Lawaetz and Stedmon, 2009; Kothawala et al., 2013). Scans were processed using a Matlab (v2007b) script written and specified by Nelson et al. (2015) and Quinlan et al. (2018); most recent version available at doi: 10.5281/zenodo.3479841), modified to additionally capture the peak present at Excitation 240 nm and Emission 300 nm (phenylalanine-like: Lakowicz, 2010). Six modeled components were validated using split half validation and outlier analysis (Quinlan et al., 2018). All PARAFAC components had similar excitation-emission maxima and strong covariation among samples with previously identified fluorophores (Quinlan et al., 2018); for subsequent analyses we examined established fluorescence maxima from the literature (Coble, 1996; Stedmon et al., 2003; Lakowicz, 2010).

#### DNA Extraction and 16S rRNA Gene Sequencing

Planktonic microbial DNA was collected by filtering 247 mL of water through 0.2 μm polyethersulfone filters (47 mm; Sterlitech) at the beginning and end of the experiment. CCA microbiome DNA was extracted at the conclusion of the experiment by cutting a fragment from each CCA plant using bone cutters rinsed with ethanol and filter sterilized water. Fragments of CCA and filters were flash frozen in liquid nitrogen and stored at –80°C until extraction. Fragments and filters were homogenized by bead beating using a Fastprep homogenizer and DNA was extracted using the MoBio Powersoil DNA extraction kit following the manufacturers protocol. The V3-V4 region of the 16S rRNA gene was amplified using polymerase chain reaction following the protocols of Kozich et al. (2013) and the 341F and 785R primers recommended by Klindworth et al. (2013). The resultant sequences were analyzed with mothur (v. 1.39.5; Schloss et al., 2009) following standardized pipelines described in Goldberg et al. (2017). Two-thousand sequences were randomly subsampled from each sample to standardize sampling effort. One replicate calcium carbonate control from each water treatment (filtered and unfiltered) and all three control water samples from the filtered water treatment could not be sequenced because of weak amplification. Sequence reads were aligned to the SILVA database (v. 115) assigned to OTUs via clustering (OptiClust; Westcott and Schloss, 2017) at a sequence identity level of 97%. OTU data were processed using a custom R script^1^.

### Data Analysis

Cell count concentrations and raw fDOM parameter Raman units were log_10_-transformed to approximate a Gaussian (normal) distribution. Mean fDOM concentrations of the water control in the respective water treatment (filtered or unfiltered) were initially subtracted from each treatment and then each treatment was standardized to CCA surface area before log_10_-transformation. No DOC measurements for water controls were collected because DOC measurements were used to compare bulk exudation by calcium carbonate and rhodoliths and as such were only standardized to surface area then log_10_-transformed. Rates of fDOM release were calculated as R.U. cm^–2^h^–1^ (Supplementary Table S1). With our experimental design we are unable to resolve which portions of the DOM pool are microbially- or organism-derived. As a result, all DOM measurements were analyzed at the final timepoint as a total day exudation comparison. Change in cell counts were calculated as the cell concentrations at 9:00 subtracted from the 17:00 time point and then log10-transformed for statistical tests and visualization. We maintain three replicates in all factorial combination of treatments which conserves statistical power within our ANOVAs and *post hoc* analyses. A two-way ANOVA was used to assess differences in DOM exudation among species or water treatment. Tukey *post hoc* tests were used to assess significance differences in DOM between species at α = 0.05. OTU relative abundances were arcsine square root transformed. A two-way ANOVA was used to assess differences in OTU relative abundances of bacterioplankton communities between *P. onkodes* and *H. reinboldii* or water treatment. A one-way ANOVA was used to assess differences in OTU relative abundances of microbiome associated communities between the two CCA species. Sequences which could not be identified to at least order level were discarded leaving 660 unique OTUs. False discovery rate (FDR) corrections were conducted on all *p*-values (Benjamini and Hochberg, 1995).

Principal coordinate analysis (PCoA) plots were used to visualize Bray-Curtis distance matrices generated from 660 observed square root and arcsine transformed OTU relative abundances. One-way PERMANOVAs were used to assess significance of differences between CCA species-facilitated microbial communities by filtration and DNA source (microbiome, bacterioplankton; seed = 100, and permutations = 1000) with Bray-Curtis distance matrices calculated from the transformed relative abundances.

## Results

### Effect of Species and Water Treatment on DOM Concentrations

**FIGURE 1:**
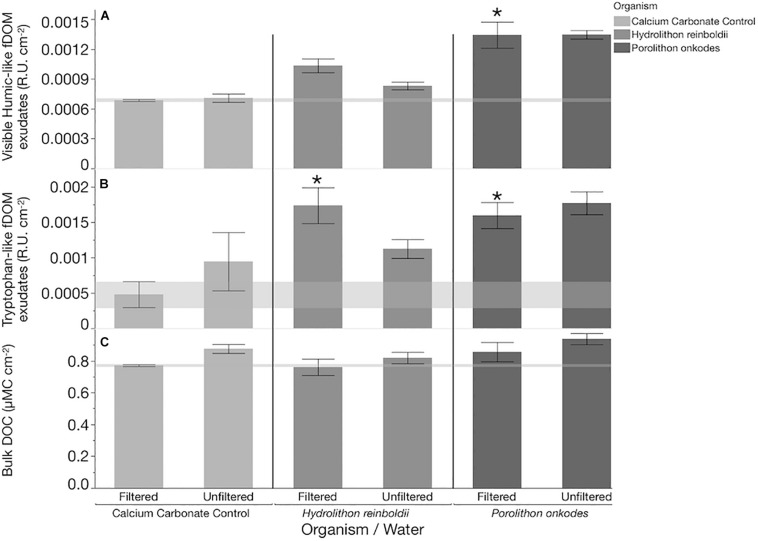
DOM concentrations within each treatment after 8 h. Concentrations of **(A)** visible Humic-like fDOM exudates, **(B)** tryptophan-like fDOM exudates, and **(C)** bulk DOC are shown as bars (mean, *n* = 3, whiskers are SE). Shaded horizontal bands bracket mean and standard error of the calcium carbonate control for each parameter to illustrate background levels of DOM in filtered water treatments. R.U. is raman units of water. All treatments have had baseline levels (mean water control) of each fDOM variable subtracted before visualization. Before subtraction, exudates from each organism in both water treatments were significantly higher than levels within the water control (Dunnett’s FDR *p* < 0.0142) except for tryptophan-like fDOM within calcium carbonate controls (Dunnett’s FDR *p* > 0.05). “^∗^” indicate treatments which had significantly higher concentrations of exudate than the respective calcium carbonate control after water blank subtraction (Tukey FDR *p* < 0.045).

In filtered water, both CCA species produced more tryptophan-like fDOM than the calcium carbonate control (Tukey FDR *P* < 0.045). In unfiltered water, *P. onkodes* and *H. reinboldii* did not significantly differ in fDOM exudate concentrations of either proteinaceous or humic-like fDOM (Figure 1; Tukey FDR *p* > 0.05) and there was no significant difference in tryptophan-like fDOM exudates between any of the treatments (calcium carbonate control, *P. onkodes, H. reinboldii*; Tukey FDR *p* > 0.05). In both filtered and unfiltered treatments, bulk DOC did not significantly differ between either CCA species or calcium carbonate control. There was no significant difference between fDOM concentrations between water treatments (Two-way ANOVA *p* > 0.05).

### Effect of Species and Water Treatment on Microbial Community

**FIGURE 2:**
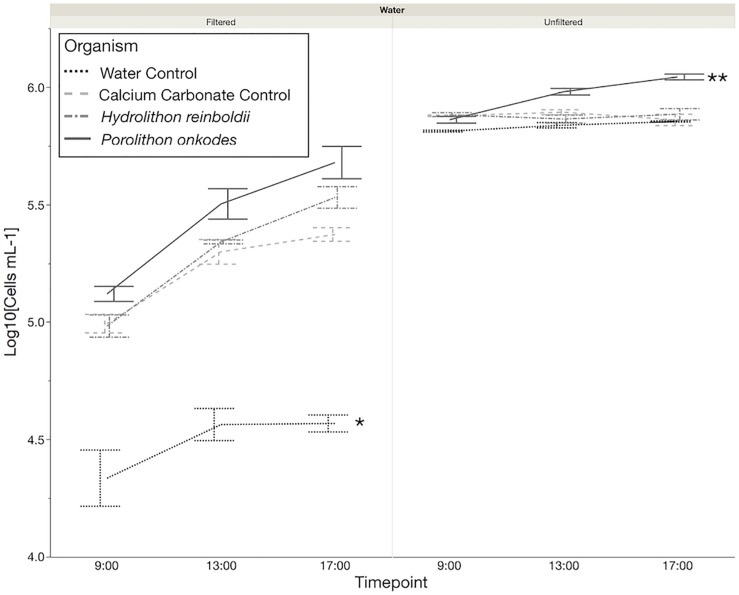
Microbial community growth over 8 h. Flow cytometry was used to quantify the cells mL^–1^, which were log10 transformed. ^∗^Water control Tukey *P* < 0.0029, Tukey *p* < 0.05. ^∗∗^*Porolithon onkodes*, Tukey *p* < 0.0199; significant differences based off of change in cells from the beginning of the incubation to the end.

In filtered water, the bacterial growth rates from both treatments of CCA species and the calcium carbonate control were larger than in the water only control (Tukey *p* < 0.005; Figure 2), but they did not differ in relation to each other. In unfiltered water, communities in association with *P. onkodes* grew (Tukey *p* = 0.0199), but neither bacterial growth rates from *H. reinboldii* nor calcium carbonate control treatments differed from the background water (Tukey *p* > 0.05).

**FIGURE 3:**
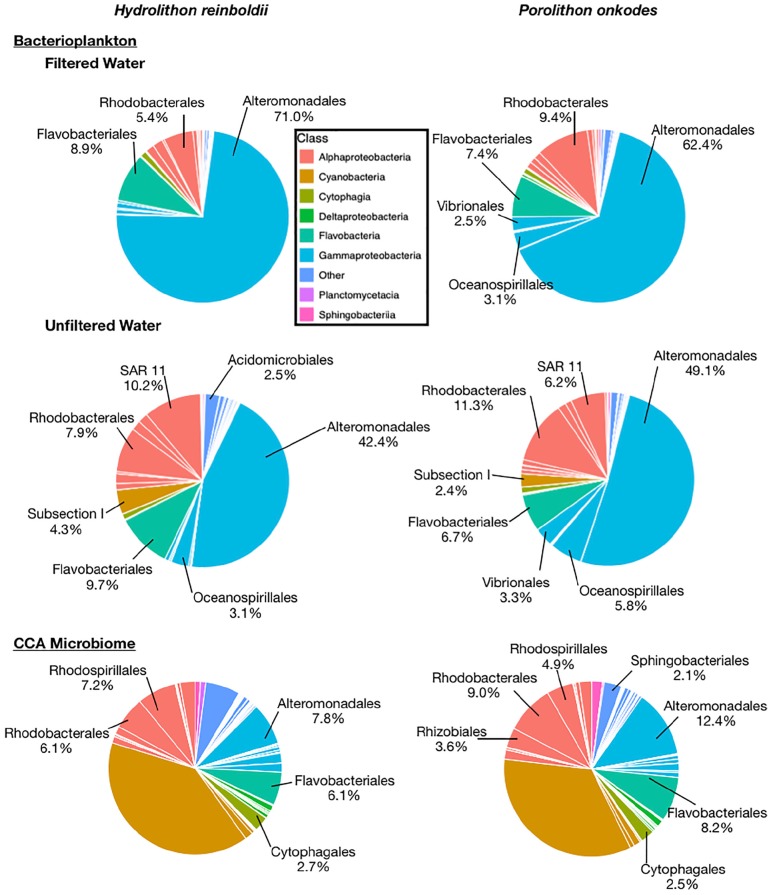
Relative abundances of microbial orders found with *Hydrolithon reinboldii* and *Porolithon onkodes.* Mean relative abundances were calculated from replicate plants, *n* = 3. Pie wedges represent orders and color fill represents class. Orders that could be classified and had relative abundances greater than 2% are labeled.

A total of 180,216 16S amplicon reads were recovered across all samples with an average of 24,515 and 39,665 reads in *H. reinboldii* and *P. onkodes* samples, respectively (Supplementary Table S2). Bacterioplankton communities across both CCA species were dominated by Gammaproteobacteria in both filtered and unfiltered treatments with an overall average of 63.4 ± 11.1%: 72.3 ± 5.9% in filtered water treatments and 54.4 ± 7.0% in unfiltered water treatments (Figure 3).

**FIGURE 4:**
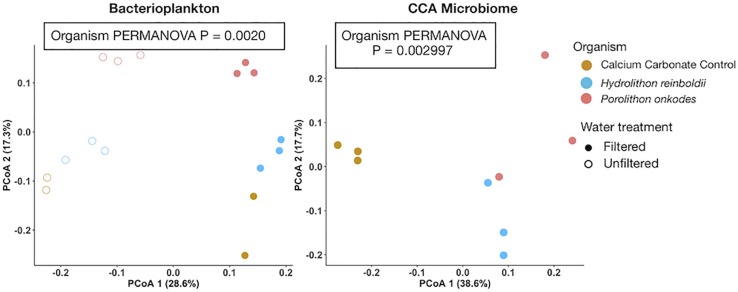
Principal coordinate analysis of bacterioplankton and microbiome community assemblages. Bray-curtis distances were calculated from arcsine transformed OTU relative abundances. Colors indicate organism and fill indicates water treatment.

Bacterioplankton communities were significantly different among organismal treatments in both filtered (PERMANOVA *p* = 0.0020; Figure 4) and unfiltered (PERMANOVA *p* = 0.0009) water treatments. Bacterioplankton associated with *P. onkodes* exhibited increased OTU richness within the filtered treatment as compared to *H. reinboldii* (*t*-Test p = 0.0334). CCA microbiomes were also significantly distinct between CCA species (PERMANOVA p = 0.002997). There was no observable difference between CCA species in other metrics of alpha diversity, including Shannon diversity or Pielou’s J evenness indices in the bacterioplankton or microbiome communities (One-way ANOVA, P > 0.05).

**FIGURE 5:**
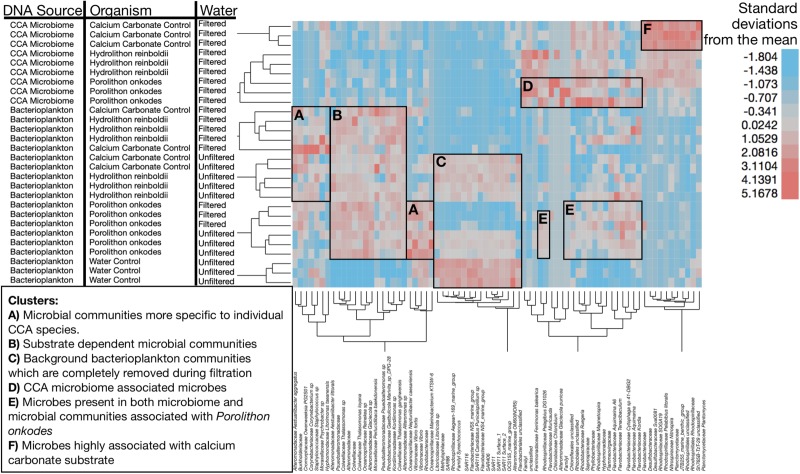
Clustering of microbial community samples by OTU. Each row is a sample and annotated to denote DNA source, water treatment and organismal treatment. Regions highlighted to show specific associations of bacteria to specific clusters of samples. Width of rectangles guided by cluster dendrogram to highlight clusters of samples with similar experimental characteristics. Height of the highlighting was selected to illustrate the organisms whose relative abundances were most enriched in those sample clusters. **(A)** Microbial communities more specific to individual CCA species. **(B)** Substrate dependent microbial communities. **(C)** Background bacterioplankton communities which are completely removed during filtration. **(D)** CCA microbiome associated microbes (boxed around Porolithon onkodes). **(E)** Microbes present in both microbiome and microbial communities associated with Porolithon onkodes, suggesting sloughing from CCA microbiome. **(F)** Microbes highly associated with calcium carbonate substrate. Heat map illustrates standardized relative abundance of OTUs in units of standard deviation. Dendrogram was generated using Ward’s minimum variance method to hierarchically cluster samples by OTU relative abundance, square root then arcsine transforming, deriving means, standard deviations and standardizing using z-scores. We used Wards’ minimum variance hierarchical clustering with input data standardized to units of standard deviation using z-scores [(x- μ)^∗^SD(x)^–1^] to visualize patterns of association among samples and taxa. For clearer visualization, hierarchical clustering was conducted only on clades which had a minimum relative abundance of 10% in at least one sample (*n* = 75 distinct OTUs).

The differences between organismal-facilitated bacterioplankton and CCA tissue microbiome populations are further illustrated within Figure 5 and statistically annotated in Table 1 (statistically larger mean treatment relative abundances are bolded). Bacterioplankton communities facilitated by CCA were different by species (Figure 5A and Table 1) and distinct from those in the input water (Figure 5B). Filtration consistently removed water-associated taxa (Figure 5C). CCA tissue microbiomes were clearly distinct from planktonic communities (Figure 5D), but a suite of bacteria associated with *P. onkodes* was present in both the CCA tissue and planktonic communities (Figures 5D,E). The microbial community inhabiting the calcium carbonate control was distinct from CCA in being dominated by a narrow set of taxa (Figure 5F). *Hydrolithon reinboldii* facilitated fewer distinct OTUs than *P. onkodes* (Table 1 and Figure 5).

**TABLE 1:** OTUs significantly induced by each CCA species and water treatment.

		**Clade**					**Relative abundances treatment mean**	
					
**DNA Source**	**Anova test**	**Order**	**Family**	**Genus/OTU**	**Anova**	**FDR *P*-value**	**Hydrolithon reinboldii**	**Porolithon onkodes**
Bacterioplankton	Organism	Alteromonadales	Alteromonadaceae	*Aliagarivorans*	>1e−4	>1e−4	0.00%	**0.06%**
		Alteromonadales	Alteromonadaceae	*Alteromonas*	0.0001	0.0074	**25.15%**	14.71%
		Alteromonadales	Alteromonadaceae	*Glaciecola*	0.0000	>e−4	1.13%	**3.47%**
		Bacteroidales	Bacteroidaceae	*Bacteroides*	0.0012	0.0476	0.00%	**0.03%**
		Flavobacteriales	Flavobacteriaceae	*Leeuwenhoekiella blandensis*	0.0010	0.0400	**0.04%**	0.00%
		Oceanospirillales	Oceanospirillaceae	*Oceanobacter*	0.0001	0.0077	0.01%	**0.11%**
		Oceanospirillales	Oceanospirillaceae	*Reinekea*	0.0003	0.0168	0.33%	**1.70%**
		Oceanospirillales	SAR86		>1e−4	0.0036	**0.69%**	0.62%
		Rhodobacterales	Rhodobacteraceae		>1e−4	0.0020	5.36%	**9.53%**
		Vibrionales	Vibrionaceae	*Vibrio fortis*	0.0009	0.0400	0.01%	**0.92%**

							**Filtered**	**Unfiltered**

	Water	Acidomicrobiales	OCS155		>1e−4	0.0037	0.03%	**1.82%**
		Alteromonadales	Alteromonadaceae	*Aliagarivorans*	0.0010	0.0400	0.00%	**0.06%**
		Alteromonadales	Alteromonadaceae	*Alteromonas*	0.0003	0.0185	**25.54%**	14.32%
		Alteromonadales	Alteromonadaceae	*OM60(NOR5)*	0.0010	0.0400	0.00%	**0.29%**
		Bacteroidales	Bacteroidaceae	*Bacteroides*	0.0010	0.0400	**0.03%**	0.00%
		Deferribacteres	SAR406		0.0006	0.0332	0.00%	**0.20%**
		Flavobacteriales	Flavobacteriaceae	*Leeuwenhoekiella blandensis*	>1e−4	0.0036	**0.04%**	0.00%
		Flavobacteriales	Flavobacteriaceae	*NS5*	>1e−4	0.0020	0.03%	**0.63%**
		Methylophilus	Methylophilaceae		>1e−4	0.0018	0.00%	**0.17%**
		Oceanospirillales	SAR86		0.0010	0.0400	0.00%	**1.31%**
		Rhodospirillales	Rhodospirillaceae	*AEGEAN-169*	0.0001	0.0049	0.01%	**1.10%**
		Rickettsiales	SAR116	*Candidates Puniceispirillum*	0.0004	0.0226	0.03%	**0.53%**
		Rickettsiales	SAR116		>1e−4	0.0036	0.03%	**0.62%**
		SAR11	Surface_1		0.0001	0.0065	0.11%	**5.15%**
		SAR_11	Surface_2		>1e−4	0.0020	0.07%	**1.91%**
		SubsectionI	Familyl	*Synechococcus*	0.0001	0.0050	0.03%	**3.28%**
	Organism * Water	Alteromonadales	Alteromonadaceae	*Aliagarivorans*	>1e−4	0.0037	—	—
		Alteromonadales	Alteromonadaceae	*Alteromonas*	0.0001	0.0090	—	—
		Bacillales	Staphylococcaceae	*Staphylococcus*	>1e−4	0.0002	—	—
		Bacteroidales	Bacteroidaceae	*Bacteroides*	0.0008	0.0394	—	—
		Flavobacteriales	Flavobacteriaceae	*Leeuwenhoekiella blandensis*	0.0003	0.0162	—	—
CCA Microbiome	Organism	Alteromonadales	Colwelliaceae	*Thalassomonas ganghwensis*	>1e−4	0.0008	0.00%	**0.05%**
		Desulfobacterales	Nitrospiraceae	*Candidatus Entotheonella palauensis*	>1e−4	0.0020	0.13%	**0.00%**
		Ignavibacteriales	IheB3-7		>1e−4	0.0001	**0.05%**	0.00%
		Oceanospirillales	OM182	HTCC2178	>1e−4	0.0013	0.00%	**0.07%**

*^∗^Bold values are significantly more abundant (Anova *p* < 0.05).*

## Discussion

Our results demonstrate that *H. reinboldii* and *P. onkodes* exude equivalent quantities of DOC but differ compositionally in exuded fDOM (humic-like and tryptophan-like fDOM; Figure 1). *P. onkodes* likely sloughs bacteria into the water (Figures 2, 5E) and the two CCA species induced distinct microbial communities across their microbiome and in the water column (Figures 3, 4).

Exudation of DOM and the facilitation of microbial communities through that exudation has been measured in relation to different primary producers across the coral reef ecosystem. Portions of the DOM pool have been shown to differ in both bulk concentration and composition between the major reef primary producers (dissolved combined neutral sugars and fDOM concentrations; Haas et al., 2013; Nelson et al., 2013; Quinlan et al., 2018). Similar to other methods used to characterize the DOM pool we are only capable of resolving a small and variable fraction of the DOM pool and infer that if a small portion of the pool differs then the unmeasured remainder of the DOM likely exhibits similar magnitudes of differentiation among producer sources (DOC, Carlson et al., 2010; DCNS, Nelson et al., 2013; fDOM Quinlan et al., 2018; LCMS, Petras et al., 2017). As reported previously by Brocke et al. (2015), and Haas et al. (2011), compared to other macro algae in coral reefs, CCA produce roughly equivalent quantity of DOC. However, when these other macro-algal species have been compared to one another, a subset of their DOM exudate pools, dissolved combined neutral sugars, are compositionally different and facilitate distinct microbial communities (Nelson et al., 2013; Wegley Kelly et al., 2018). Similar comparisons of dissolved organic matter exudation have not been measured between different species of CCA until now. The findings presented here begin to parse the differences in CCA species exudate composition (Figure 1) and their role in the facilitation of different microbial communities (Figure 5).

Bulk DOC measurements did not differ between calcium carbonate controls and CCA, however, this is likely as a result of the release/dissolution from endoliths/calcium carbonate of the controls. The differences we can resolve between calcium carbonate and algal species are most notable in fDOM composition. Humic-like and tryptophan-like fDOM visually appear to be degraded by planktonic microbes in the unfiltered treatment of *H. reinboldii* (Figures 1A,B), however, there was no statistical difference in treatments. This could be a result of the low resolution of fDOM analyses. However, both *Alteromonas* sp. and *Leeuwenhoekiella blandensis* were more abundant in filtered water exposed to *H. reinboldii* (Table 1). This suggests that they are outcompeted by more common bacterioplankton, but were removed by the filtration.

Our amplicon sequencing results support (Sneed et al., 2015) finding that the CCA microbiome communities were significantly different between *Hydrolithon* and *Porolithon* genera, even though we tested different species from the Pacific Ocean (Figure 5 and Table 1). This experiment was designed to resolve directly the effects of CCA on the planktonic community, clarifying that this effect is due to the CCA and not simply the carbonate skeleton. Notably, community structures of bacterioplankton associated with each CCA were similar whether water was filtered or not (Figure 5A), suggesting that much of the influence is through direct sloughing or rapid growth of CCA associated microbes (Figure 5B) rather than enrichment of abundant planktonic taxa (Figure 5C).

*Porolithon onkodes* exudates selected for clades of bacteria that have been associated more commonly with coral disease, such as *Rhodobacteraceae* and *Vibrio fortis* (Table 1). Although, Rhodobacteraceae is frequently found in biofilm communities (Dang and Lovell, 2016), Rhodobacteraceae has previously been identified as a possible agent of *Porites* white patch syndrome (Séré et al., 2013). Although *Vibrio fortis* has not been described as a potential pathogen, it is related to OTUs possessing virulent homolog (Persson et al., 2009). Although we have found plausible agents of coral disease, we do not directly show that these microbial communities would cause the diseases they have been associated with in prior studies.

Some of the OTUs which were found in the CCA microbiome were also found in the bacterioplankton communities of *P. onkodes* (Figure 5E). This suggests that the clades such as *Glaciecola* sp. were sloughed from the *P. onkodes* microbiome into the water. This finding would explain the increase in bacterioplankton communities observed within filtered treatments (Figure 2) and agrees with previous findings that *P. onkodes* sloughs its outer tissue layer as an antifouling mechanism (Harrington et al., 2004). Both the presence of plausible agents of disease and the tissue sloughing could reduce settlement and survivorship of coral larvae.

In contrast, exudates of *H. reinboldii* facilitated the growth of bacteria associated with antimicrobial activity such as *Alteromonas* spp., which has been cultured from marine environments and produces the antimicrobial compound, Thiomarinol (Shiozawa et al., 1993). In fact this bacterial taxon was also found in *Hydrolithon boergesenii* from the Caribbean (Sneed et al., 2015) *Alteromonas* spp. can induce high rates of larval settlement in other marine invertebrates (Huggett et al., 2006). The mechanism for the induction of larval settlement as a result of *Alteromonas* is largely unknown. However, metamorphosis-associated contractile structure (MAC) genes have been investigated within the genus *Pseudoalteromonas* for their effect on invertebrate larval metamorphosis and settlement (Shikuma et al., 2014). To determine whether *Alteromonas* has MAC genes, further metagenomics analyses would have to be conducted. Along with MAC structures, other research has proposed the presence of specific microbially produced chemicals such as tetrabromopyrrole that induce the metamorphosis of invertebrate larvae (Tebben et al., 2011). Our study did not conduct high enough resolution DOM analyses to observe the production of such compounds, however, *Pseudoalteromonas* was found across both *H. reinboldii* (bacterioplankton mean abundance = 0.51 ± 0.33%, microbiome mean abundance = 0.00 ± 0.00%) and *P. onkodes* (bacterioplankton mean abundance = 0.38 ± 0.2 6%, microbiome mean abundance = 0.15 ± 0.15%) but did not differ statistically between species or from the water control (mean = 0.06 ± 0.12%). These findings are striking since even between widely separated ocean basins, the Caribbean (Sneed et al., 2015) and Pacific, *Hydrolithon* spp. facilitate similar and specific clades of bacteria that are not commonly found in opportunistic and pathogenic biofilms. These less opportunistic microbiomes could provide a more hospitable settlement environment for larvae.

One possibility is that the different species of CCA control their bacterial communities through either periodic sloughing or the facilitation of antimicrobial clades regardless of the DOM they produce. These CCA species would then host a biofilm community of either primary settlers or more similar to a climax community, respectively. This hypothesis suggests that coral larval settlement is inhibited by tissue sloughing and facilitated by climax microbial communities, which is consistent with research showing that coral larvae preferentially settle on older biofilms (Webster et al., 2004).

## Conclusion

In conclusion, we found that *P. onkodes* released microbes into the seawater and facilitated the growth of bacterial OTUs that might cause mortality for coral larvae. Conversely, *H. reinboldii* facilitated a community of microbes that can have more antimicrobial properties and were previously associated with invertebrate larval settlement. These results help to clarify that different species of CCA growing in the same habitats host distinct surface microbiomes and can impact the local seawater microbial communities by differential fDOM production and the sloughing of tissue. Further investigation into the metagenomics of the microbial communities, the structure of the DOM produced by these, and other CCA species and how these induced bacterial communities help or inhibit coral larvae before and after settlement are warranted.

## Data Availability Statement

Fluorescent dissolved organic matter, dissolved organic carbon and flow cytometry data have been uploaded to BCO-DMO (https://www.bco-dmo.org/project/675025). Raw microbial sequences have been uploaded to NCBI under the accession PRJNA578682. All data and R code has also been made available on Github, doi: 10.5281/zenodo.3479841.

## Author Contributions

RR-W collected the *Hydrolithon reinboldii* and *Porolithon onkodes* for this experiment under Hawai‘i Department of Land and Natural Resources Division of Aquatic Resources Special Activities Permit 2015–2017 to the Hawai‘i Institute of Marine Biology. RR-W, ZQ, and BC identified the two species of CC. ZQ designed, setup, and ran the experiment, ran the fluorescent dissolved organic matter and flow cytometry samples, and analyzed all data with support from CN. BC extracted the DNA. All authors contributed to the writing of the manuscript.

## Conflict of Interest

The authors declare that the research was conducted in the absence of any commercial or financial relationships that could be construed as a potential conflict of interest.

## Footnotes

1 https://github.com/Zquinlan/R_Scripts/blob/master/pipelines/microbial/CRUSADE_analysis.R
