# Supplementary material for: Species-Specific Differences in the Microbiomes and Organic Exudates of Crustose Coralline Algae Influence Bacterioplankton Communities
Source: Front Microbiol. 2019 Nov 8;10:2397. doi: 10.3389/fmicb.2019.02397 (PMC6857149; doi:10.3389/fmicb.2019.02397)
Supplement: Supplementary file 1 [file Table_1.docx]

| Supplemental Table 2.Total number of sequences from each organismal treatment | | | | | |
| --- | --- | --- | --- | --- | --- |
|  |  | *Average number of Sequences per sample* | |  | *total number of sequences* |
| *Water treatment* |  | *Microbiome* | *Bacterioplankton* |  | *Bacterioplankton* |
| Filtered | Background Water |  | 1,126 |  | 3,378 |
|  | Calcium Carbonate Control | 24,223 | 4,810 |  | 14,430 |
|  | *Hydrolithon reinboldii* | 33,867 | 8,630 |  | 25,891 |
|  | *Porolithon onkodes* | 42,363 | 14,135 |  | 42,404 |
| *Unfiltered* | Background Water |  | 5,293 |  | 15,879 |
|  | Calcium Carbonate Control |  | 6,056 |  | 18,167 |
|  | *Hydrolithon reinboldii* |  | 7,713 |  | 23,140 |
|  | *Porolithon onkodes* |  | 12,309 |  | 36,927 |
| Total |  |  |  |  | 180,216 |

| Supplemental Table.1 Rates of fDOM release | | | | | | |
| --- | --- | --- | --- | --- | --- | --- |
| Water | Organism | Fulvic Acid-like  (R.U. cm-^2^ h^-1^) | Marine Humic-like  (R.U. cm-^2^ h^-1^) | Ultra Violet Humic-like  (R.U. cm-^2^ h^-1^) | Visible Humic-like  (R.U. cm-^2^ h^-1^) | Tryptophan-like  (R.U. cm-^2^ h^-1^) |
| Filtered |  |  |  |  |  |  |
|  | Calcium Carbonate Control | 3.270E-05 | 7.000E-05 | 6.876E-05 | 6.782E-05 | 5.816E-05 |
|  | *Hydrolithon reinboldii* | 4.429E-05 | 7.865E-05 | 7.960E-05 | 9.611E-05 | 3.871E-05 |
|  | *Porolithon onkodes* | 6.087E-05 | 1.093E-04 | 1.012E-04 | 1.265E-04 | 5.938E-05 |
| Unfiltered |  |  |  |  |  |  |
|  | Calcium Carbonate Control | 4.112E-05 | 6.618E-05 | 6.846E-05 | 6.482E-05 | 4.106E-05 |
|  | *Hydrolithon reinboldii* | 3.849E-05 | 6.688E-05 | 6.973E-05 | 7.736E-05 | 1.042E-05 |
|  | *Porolithon onkodes* | 6.649E-05 | 1.032E-04 | 1.086E-04 | 1.281E-04 | 1.287E-04 |
